# The Diagnosis of Autism: From Kanner to DSM-III to DSM-5 and Beyond

**DOI:** 10.1007/s10803-021-04904-1

**Published:** 2021-02-24

**Authors:** Nicole E. Rosen, Catherine Lord, Fred R. Volkmar

**Affiliations:** 1 University of California, Los Angeles, USA; 2 Yale University, New Haven, USA; 3 Southern Connecticut State University, New Haven, USA

**Keywords:** Autism, History, Dimensional, Categorical, DSM

## Abstract

In this paper we review the impact of DSM-III and its successors on the field of autism—both in terms of clinical work and research. We summarize the events leading up to the inclusion of autism as a “new” official diagnostic category in DSM-III, the subsequent revisions of the DSM, and the impact of the official recognition of autism on research. We discuss the uses of categorical vs. dimensional approaches and the continuing tensions around broad vs. narrow views of autism. We also note some areas of current controversy and directions for the future.

It has now been nearly 80 years since Leo Kanner’s (1943) classic description of infantile autism. Official recognition of this condition took almost 40 years; several lines of evidence became available in the 1970s that demonstrated the validity of the diagnostic concept, clarified early misperceptions about autism, and illustrated the need for clearer approaches to its diagnosis. As a result of this work, autism was included for the first time in DSM-III (APA 1980) and maintained in every subsequent edition of the manual. That said, the definition of autism has in fact alternated over time between broader and narrower views of the condition. Throughout this review, we will discuss the evolution of autism as a diagnostic concept as well as highlight important areas of work on the condition including the impact of gender, culture, social class, race/ethnicity, age, and cognitive ability that continue to be the focus of research.

In undertaking this review, we are aware that terms have shifted over time. Not surprisingly, the name of the condition first described by Kanner has changed across the past few decades. When we refer to the concept in general, we will use the term autism, and when we refer to particular, earlier diagnostic constructs, we will use more specific terms like autism spectrum disorder, infantile autism, and autistic disorder. This issue of changes in terminology also arises with respect to Asperger’s disorder and the broader autism phenotype; in general, we will try to separate these terms to make it clear that they are not necessarily synonymous.

## Autism Before DSM-III

Any discussion of the development of autism as a diagnostic concept inevitably starts with the work of Leo Kanner and his landmark observation in 1943 (Kanner 1943). Kanner (1943) described 11 children, 8 boys and 3 girls, who presented with “inborn autistic disturbances of affective contact”. He emphasized two essential features of the condition: (1) autism—or severe problems in social interaction and connectedness from the beginning of life, and (2) resistance to change/insistence on sameness. The latter term also included some of the unusual stereotyped movements he noted such as body rocking and hand flapping. To Kanner, these movements appeared to be ways for the child to maintain sameness in his/her world. Kanner’s report was, of course, groundbreaking, but it is also important to note that even earlier descriptions of children who likely had autism were made in the 1800s in a training school for the intellectually disabled (Donvan and Zucker 2016) and in the 1700s with some reports of feral children (Candland 1995). Presumably, these feral children had either been abandoned or run away from their parents, the latter being a problem still noted by families of children with autism today (Anderson et al. 2012).

Although attracting considerably less attention at the time, Hans Asperger’s report (Asperger 1944) of boys who had marked social difficulties, unusual circumscribed interests, and good verbal skills was also monumental. While the independence of Kanner’s and Asperger’s observations are debated, with some historians suggesting that Kanner may have been aware of Asperger’s work prior to publishing his 1943 report (Silberman 2015), both men described different conceptualizations of autism that uniquely contributed to our understanding of the disorder today. Unlike Kanner who emphasized the importance of autism as a developmental condition, Asperger described behaviors that more closely resembled a personality disorder and reported that fathers of his cases showed similar problems. In an important way, Asperger’s report started what has been a continued debate on the boundaries of autism, the “broader autism phenotype”, and issues of neurodiversity (Ingersoll and Wainer 2014; Silverman 2015).

Kanner’s report was remarkably clear. He noted many of the features we still encounter in work with individuals with autism. These include things like echolalia, pronoun reversal, and unusual prosody. As we will discuss later, the relationship of problems in communication (more broadly defined than just problems in language) has been an important consideration over time, as has our understanding of the ‘insistence on sameness’ principle noted by Kanner in 1943.

As much as we are grateful for his clinical insight, Kanner’s report also contained some potential false leads for early research. Among the most important was that his use of the word autism immediately called to mind impaired self-centered thinking of the type noted by Bleuler as one of the characteristics of schizophrenia (Bleuler 1911). Others interpreted Kanner’s findings as suggesting that autism might be the earliest form of that condition. Additionally, as we discuss below, Kanner’s suggestion that autism was not associated with other medical conditions proved incorrect (Rutter and Thapar 2014; Yuen et al. 2019).

During the 1970s, there were important developments in the area of psychiatric diagnosis in general and in autism in particular that contributed to the decision to include autism as an official diagnostic category. Overall, there was increasing dissatisfaction with the chaotic state of affairs that had prevailed with psychiatric diagnoses in earlier versions of the APA’s manual. The guidelines had been heavily theoretical, were of little use for research, and had limited applicability, particularly for children. For example, in DSM-II (APA 1968), only the category of childhood schizophrenic reaction was available to describe individuals with early childhood onset of severe disturbances in development of the type referred to by Kanner in his 1943 report. This state of affairs began to change with the advent of the research diagnostic criteria (RDC) approach of the Washington University School of Psychiatry in Saint Louis (Spitzer et al. 1978; Woodruff et al. 1974). Also important was a growing awareness of the need to represent the range of difficulties that patients, particularly children, experience in other areas such as developmental and medical problems (Rutter et al. 1969).

For autism, several important developments occurred in the latter half of the 1960s and during the 1970s related to defining and diagnosing autism. As discussed later, Rimland (1964, 1968) created the first checklist for assessing symptoms suggestive of autism. Several lines of research converged to suggest that autism was a distinctive concept in its own right and not the earliest manifestation of schizophrenia. Thus, Rutter (1978) proposed a new definition of autism that included delayed and deviant social and language abilities beyond general developmental level, as well as restricted interests and repetitive behaviors—all with onset early in life. This definition proved highly influential in the advent of DSM-III. The American National Society for Autistic Children (NSAC 1978) also proposed a definition that included unusual rates and sequences of development (which overlapped to some degree with Rutter), but also emphasized hypo- and hyper-sensitivities to the environment. Although less influential for DSM-III, sensory sensitivities in autism have long been recognized and now, almost 40 years later, have been included in DSM-5 (see subsequent discussion).

Several lines of research were critical in helping to establish the validity of autism as a diagnostic concept in DSM-III. Firstly, studies of the clinical phenomenology of autism including age of onset (for autism in early childhood) and family history of schizophrenia (not common in autism), as compared to childhood schizophrenia, made it clear that these were distinct concepts (Kolvin 1971, 1972; Rutter 1972). These concepts were further differentiated by research on treatment differences that suggested that children with autism seemed to respond better to structured teaching approaches compared to the unstructured psychotherapy approach used in schizophrenia treatment in the 1950s and 1960s (Bartak and Rutter 1973). Additionally, autism was noted to clearly be a brain-based disorder given its frequent association with epilepsy, often of adolescent onset (Volkmar and Nelson 1990). Autism was also found to be strongly genetic with higher rates of concordance in monozygotic as opposed to same sex dizygotic twin pairs (Folstein and Rutter 1977); this finding discredited Bettelheim’s “refrigerator mother” theory of autism (Bettelheim 1967) and provided support for the biological origin of autism. By 1971, this journal was established as the first devoted specifically to autism, with Leo Kanner named as its editor. The original name for the journal also included the words “childhood schizophrenia”—a term that was later dropped as it became increasingly clear that autism was a distinct condition in its own right (Schopler et al. 1979). As a result of these considerations, the decision was made to include autism (“infantile autism”), for the first time, as an official diagnostic category in the groundbreaking third edition of the Diagnostic and Statistical Manual (DSM-III; APA 1980).

## Autism in DSM-III

Autism was included in DSM-III (APA 1980) in an entirely new ‘class’ of conditions—the Pervasive Developmental Disorders (PDDs). The definition provided for “infantile autism” in DSM-III was true to the name of the disorder, emphasizing characteristics of young children. The criteria described pervasive lack of social responsiveness consistent with Kanner’s first description of the condition. However, it was also clear that individuals with autism did change over time, not always continuing to exhibit this more ‘classic’ infantile form of the disorder; thus, an additional diagnostic term, “residual infantile autism”, was included for cases that had once met criteria but no longer did so. Another diagnostic category, and its residual equivalent, were also included to describe children who had an onset of something like autism after a substantial period of normal development. It is likely that this reflected an awareness of the small handful of children in samples like that of Kolvin (1971) who developed autism after age 3. It unintentionally overlapped with the much older concept of Heller (1908). Finally, as in all of DSM-III categories, a ‘subthreshold’ concept (atypical PDD) was included for cases in which strict criteria for a specific PDD were not met but the case seemed best included in the class. This group had its own complexities given previous work on concepts like atypical personality development and what would come, over time, to be seen as the broader autism phenotype (Ingersoll and Wainer 2014).

By the time autism was first included in the DSM, several lines of research had seen serious expansion, leading to autism being considered one of the best examples of a “disorder” in child psychiatry. For example, unlike many child disorders, autism was not easily confused with extremes of “normalcy” (Rutter and Garmezy 1983). At the time that autism was first recognized in DSM-III, it appeared to be a rare disorder with a rate of 3 in 10,000 children in one of the first studies (Treffert 1970), and estimated as somewhat higher but still rare, 7 in 10,000 children, in 1999 (Fombonne 1999). A marked gender difference was also noted in that males were much more likely (3–5 times) to have the condition (Fombonne 1999). The first studies about course and outcome in autism tended to paint a rather bleak picture with relatively few individuals attaining adult self-sufficiency and independence (Howlin 1997). Nevertheless, clinicians recognized that participants in the earliest studies had typically been diagnosed later and had not had access to newer and presumably more effective interventions, so there was generally more hope for the future.

Despite the major advantage that DSM-III offered by providing official recognition of infantile autism, its problems quickly became clear. The definition itself was monothetic (i.e., all criteria must be met), potentially making the criteria less flexible. The lack of a developmental orientation to the diagnosis was problematic, with the problems of adults with autism not given adequate representation with the term ‘residual’. The rationale for the childhood onset PDD (COPDD) category was not clearly articulated, and the term Pervasive Developmental Disorders itself was cumbersome. The relationship of the broad group of cases of atypical PDD (Towbin 1997) to the more strictly defined autism was of much interest, an interest that has continued to increase as the genetic complexities of autism have begun to emerge (Rutter and Thapar 2014; Yuen et al. 2019). Despite these limitations, the impact of the recognition of autism (or “infantile autism” as it was actually termed) in DSM-III is not to be underestimated. In 1979, before autism appeared as a category in DSM, a Medline search revealed that there were approximately 128 papers on the topic. By 1985, there were 335 and by 2015, 885 research papers had appeared. This dramatic increase in research interest is no small tribute to the impact of DSM-III and its influence on the field.

## From DSM-III to DSM-IV

Though the explicit recognition of autism as a disorder in DSM-III was a major advancement, problems quickly became apparent as stated earlier. Several important changes were, accordingly, considered in the 1987 revision of DSM—the DSM-III-R (APA 1987). A significant conceptual change in DSM-III-R was the move from “infantile autism” to “autistic disorder” as the name for the condition. This change reflected an awareness of the need for a more flexible and developmentally-oriented approach that would be useful across ages and developmental levels (Siegel et al. 1988; Waterhouse et al. 1993). In many respects, this approach mirrored the recommendations of Lorna Wing for a broader view of the diagnostic concept (Wing 1993).

In DSM-III-R, a new polythetic set of 16 detailed criteria was provided. The criteria were organized into what had become the standard three major domains of dysfunction observed in autism, i.e., (1) qualitative impairments in reciprocal social interaction, (2) impairments in communication, and (3) restricted interests/resistance to change and repetitive movements. In the DSM-III-R approach, a diagnosis of autistic disorder required a total of at least eight positive criteria, two from the social domain and at least one from each of the other two categories of difficulty.

A field trial was conducted to help clarify scoring rules (Spitzer and Siegel 1990). However, this field trial was complicated by a comparison group of children with conduct disorders, not generally considered an appropriate comparison for autism. Given DSM-III-R was created to account for developmental change and developmental level as well as to provide greater clinical flexibility (Volkmar et al. 1992b), the ‘residual’ or ‘subthreshold’ category was labeled pervasive developmental disorder not otherwise specified (PDD-NOS), with no other conditions included in the PDD class (Towbin 1997). However, research quickly began to suggest that the concept of PDD may have been overly broadened (Factor 1989; Hertzig et al. 1990; Volkmar et al. 1992a).

The World Health Organization’s International Classification of Diseases, 10th edition (ICD-10; World Health Organization 1992a) adopted a rather different overarching approach with two diagnostic guides—one for clinical work (World Health Organization 1992b) and the other for research (World Health Organization 1993). In ICD-10, the decision was made to explicitly recognize other disorders, including Asperger syndrome, Rett’s disorder, and childhood disintegrative disorder (Volkmar et al. 2014). The potential for divergent United States (DSM) and international (ICD-10) views threatened to complicate research comparisons across countries and international collaborations on issues like genetic and epidemiology where agreement on diagnostic standards is particularly important. These issues were given serious consideration, and major revisions were undertaken to develop the fourth edition of DSM (DSM-IV; APA 1994).

The process for drafting DSM-IV was more elaborate than with previous versions of DSM. It included a series of work groups focused on a range of topics, a series of commissioned literature reviews and data reanalyses, and eventually a field trial done in conjunction with the ICD-10 work group (Volkmar et al. 1994). Various issues were addressed right from the start. Several of the commissioned data reanalyses suggested that, as compared to ICD-10 draft criteria, the DSM-III-R approach was overly broad. The inclusion of new categories in ICD-10, particularly Asperger’s disorder, was controversial. A large number of rather disparate approaches to the diagnosis of this condition had arisen and there was not a clear consensus on best approaches to diagnosis (Ghaziuddin et al. 1992; Gillberg and Gillberg 1989; Klin et al. 1995; Szatmari et al. 1986). An additional issue was that the ICD-10 research definition was more detailed than might be desired for usual clinical work, and the clinical definitions were somewhat vague; thus, a question was raised whether a compromise might be achieved in DSM-IV with a good balance of clinical and research consideration.

For DSM-IV, a field trial (Volkmar et al. 1994) was intended to address at least some of these issues. This large, yearlong effort was international in scope with nearly 1000 cases (all of which had some condition that would include autism in its differential diagnosis) and a number of raters and clinical sites. Both historical and contemporary information was usually available to the examiners who provided detailed ratings of various potential diagnostic criteria.

The field trial results suggested that DSM-III-R was overly broad in comparison to other systems. While the rather detailed draft ICD-10 research definitions worked well, it appeared that they could be streamlined and made compatible with the draft DSM-IV criteria. Also of note was that agreement among less experienced clinicians improved using the draft DSM-IV criteria compared to DSM-III-R. Furthermore, factor analyses produced several potential models including the traditional three-factor solution group, a two-factor (social/communication and restricted behaviors) group, and a five-factor (social, communication, restricted interests, stereotyped mannerisms, and adherence to routine) group. Given the structure of ICD-10, the decision was made to continue to use the traditional three-category model in DSM-IV with a final set of criteria that were less numerous and detailed. In addition, the inclusion of a separate diagnosis of Asperger’s disorder was supported by results from a set of 50 participants with previously well-documented cases of Asperger’s disorder who were found to differ from both participants with autism and participants with PDD-NOS. Asperger’s disorder represented an area of particular controversy and edits of the diagnostic criteria were made in the final production process beyond what was finally decided by the official DSM-IV committee.

## Categorical Approaches to DSM-5 and ICD-11

### Value of Subcategories Versus Dimensions

Building on DSM-IV and decades of research, DSM-5 (APA 2013) marks an important shift in the conceptualization of autism from a multi-categorical diagnostic system to a single diagnosis based on multiple dimensions. This change follows a history of largely unsuccessful attempts to categorize the heterogeneity of autism into empirically-defined subcategories (Charman et al. 2011; Georgiades et al. 2013; Ingram et al. 2008). DSM-IV’s diagnostic subcategories (autistic disorder, Asperger’s disorder, pervasive developmental disorder not otherwise specified (PDD-NOS), Rett’s disorder, and childhood disintegrative disorder) were located within the Pervasive Developmental Disorders (PDDs) classification. The shift to consolidation within DSM-5 was driven by findings from multiple studies that showed (1) variability in the number and severity of ASD symptoms within and between diagnostic subgroups with similar core symptom profiles (Fernell et al. 2010; Macintosh and Dissanayake 2004; Ozonoff et al. 2000; Snow and Lecavalier 2011); (2) poor predictive power of subcategories on later outcomes (Szatmari et al. 2003, 2009); (3) poor diagnostic clarity resulting in limited reliability in assigning subcategory diagnoses (Lord et al. 2000, 2012a; Walker et al. 2004); and (4) restrictions on treatment eligibility and coverage based on subtypes. The elimination of subcategories was controversial for various reasons, including concerns over the removal of an important part of an individual’s identity and community, specifically related to Asperger’s disorder, as well as concerns over losing services due to an individual no longer meeting more stringent diagnostic criteria. However, the evidence for the existence of subcategories within ASD has continued to be very weak (Miller and Ozonoff 1997, 2000). Furthermore, the shift from multiple subcategories to a single dimension resulted in improved diagnostic specificity and good diagnostic sensitivity, with over 90% of children with PDDs meeting DSM-5 ASD criteria (Huerta et al. 2012; Mandy et al. 2012), and with the remainder likely captured by the new social communication disorder diagnosis.

DSM-5 and ICD-11 (World Health Organization 2018) both utilize ASD as the unitary classification of core symptoms, though the systems differ in their approaches to describing within-group differences. To capture individual variation, alongside an ASD diagnosis, DSM-5 provides core symptom domain severity levels based on the level of support needed for individual functioning, in addition to specifiers which offer descriptions of common co-occurring non-ASD impairments (i.e., intellectual impairments, language deficits, medical and psychiatric conditions, etc.). Of note, while the concept of functionality through severity levels is important, the severity metric has shown questionable validity (Lord et al. 2012a, 2018).

Though ICD-11 also adopted ASD as the umbrella term, it retained a multi-categorical system to differentiate individuals along the spectrum with varying levels of history (i.e., regression) and intellectual and language abilities. ICD-11 contains eight subcategories of ASD diagnoses, each describing a profile of similar ASD deficits accompanied by variable combinations of intellectual and language impairments. Similar to DSM-5, ICD-11 also provides specifiers for non-ASD co-occurring medical and psychiatric conditions.

### Use of Principles Versus Examples

DSM-IV and ICD-10 criteria had included examples, derived from multiple levels of analyses, that described specific behaviors, such as shared enjoyment, general qualities, and important contexts (e.g., peer interaction), through which deficits in ASD could reliably be seen (Mahjouri and Lord 2012). Recognizing the myriad behavioral presentations among individuals with ASD of varying developmental levels, DSM-5 and ICD-11 introduced broad principles in place of specific examples to better define symptom subdomains. The new principles, each accompanied by a non-exhaustive list of similar examples, present deficits within each subdomain that are applicable across age ranges and developmental levels, thus providing greater systematic sensitivity and specificity. Notably, however, while conceptualized through clinical observation, the DSM-5 and ICD-11 criteria included within each domain are not empirically-defined dimensions (Lord and Jones 2012).

### Three-Domain Versus Two-Domain Symptom Model

The evolution of DSM-IV and ICD-10 to DSM-5 and ICD-11 also involved a restructuring of the three-domain symptom model into a two-domain symptom model by combining the communication and social symptom categories into a single social–communication domain. The restricted and repetitive interests/behaviors (RRBs) domain was maintained as separate. This change was driven by (1) a number of factor analytic findings supporting a single social–communication factor (Gotham et al. 2007; Robertson et al. 1999); (2) the somewhat arbitrary nature of categorizing specific behaviors as social or communicative given the significant overlap (Gotham et al. 2007); and (3) the lack of diagnostic specificity of structural language deficits (i.e., in vocabulary and grammar) in ASD (Bishop and Norbury 2002; Baird et al. 2008). Behavioral examples within the previous communication domain were largely incorporated into principles within DSM-5’s and ICD-11’s broadened symptom domains, such that impaired initiation/continuation of conversation and imaginative play, as well as stereotyped language, were reassigned to the social–communication and the RRBs domains, respectively. The updated factor structure of symptomatology, compared to the previous three-domain model, resulted in increased sensitivity with minimal reduction in specificity (Frazier et al. 2012).

### False Dichotomy Between Categorical and Dimensional Approaches

**Fig. 1:**
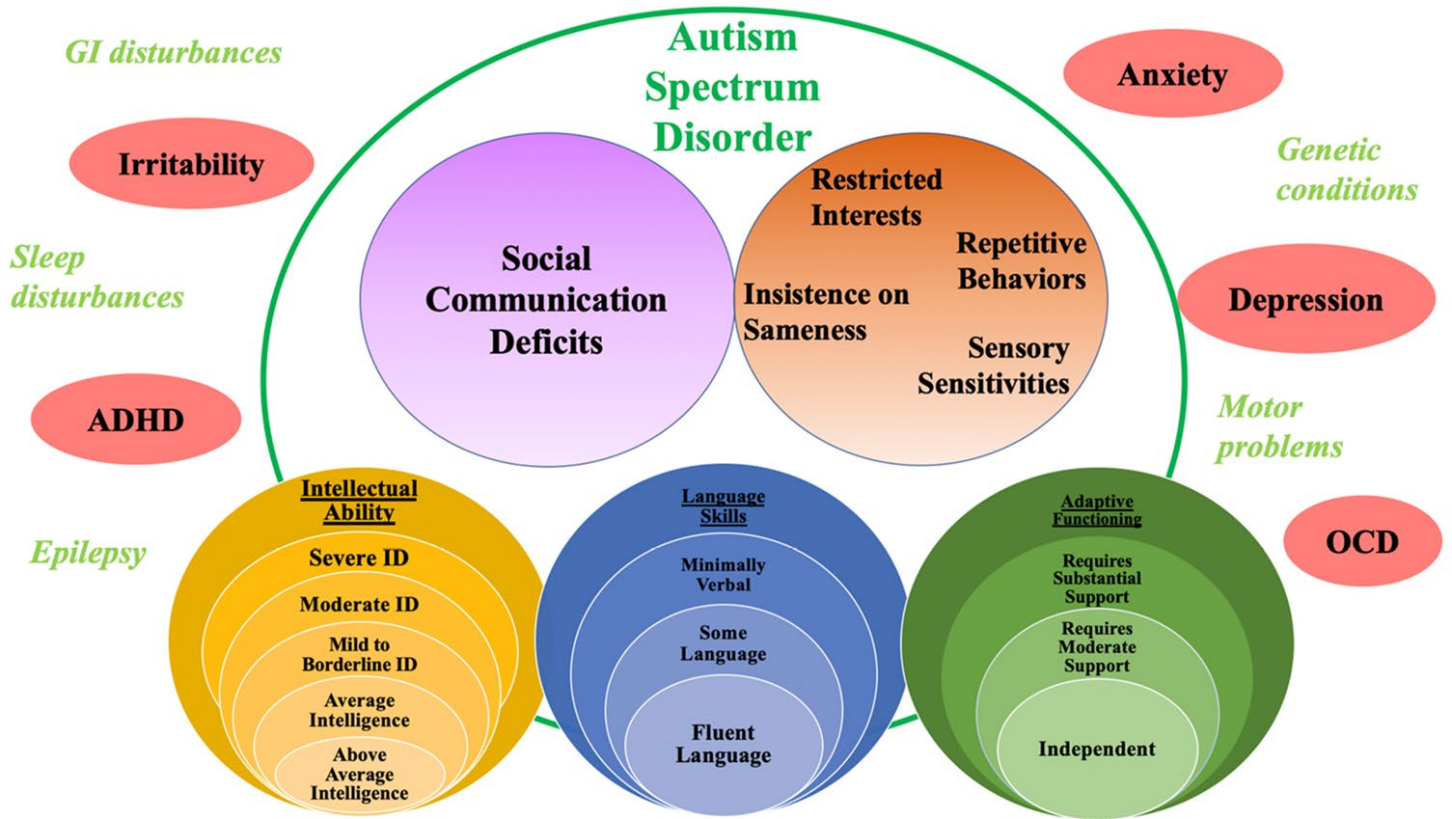
Overlap between categories and dimensions for core ASD symptoms and non-ASD symptom specifiers

Many argue that categorical and dimensional approaches are fluid, such that dimensions can become categories by defining thresholds, and categories can become dimensions by combining constructs to allow for common core features with accompanying variation, as is seen in the transition from DSM-IV to DSM-5 (see Fig. 1; Pickles and Angold 2003; Lord and Jones 2012). In the end, it may be helpful to conceptualize ASD as a single diagnostic condition consisting of various categories of symptoms that can be evaluated in terms of dimensional severity (Pickles and Angold 2003), where we choose to emphasize the dimensionality for some purposes (e.g., research and understanding mechanisms) and the category for others (e.g., practical issues related to service allocation or planning).

## Dimensional Approaches

### History of Dimensional Approaches

Dimensional approaches to the diagnosis and classification of autism can be traced back many decades. Years before autism was formally recognized in DSM-III as a disorder distinct from schizophrenia, researchers attempted to quantify the symptom profiles of individuals demonstrating the unusual patterns of behaviors as described by Leo Kanner. As research and clinical practice in the field grew, so did the diagnostic measures designed to capture the symptoms. Remarkably, almost 60 years after the introduction of the first autism diagnostic tools, current gold-standard practices largely retain many of the components of earlier versions.

One of the first widely circulated measures for assessing autism was the Diagnostic Checklist for Behavior-Disturbed Children (Forms E-1 & E-2; Rimland 1964, 1971). While Rimland’s Diagnostic Checklist is largely rooted in Kanner’s and DSM-II’s conceptualization of autism as a form of childhood schizophrenia, its emphasis on assessing the core symptoms of autism remains a fundamental component of standard practice today (Corsello 2013). Building on Rimland’s foundational measure, the Behavior Rating Instrument for Autistic and Atypical Children (BRIAAC; Ruttenberg et al. 1966) was introduced and provided the first attempt to identify autism based on a clinician’s direct observation of behavior (Corsello 2013). The use of clinician case notes to inform diagnostic decisions, rather than reliance exclusively on parent-report, improved the precision of diagnoses and advanced the field of behavior-based assessment (Corsello 2013; Parks 1983). Further refining behavior-based measures, the Brief Observation System (BOS; Freeman et al. 1978) was introduced with an emphasis on standardizing the environment and the behavior of the child during a diagnostic evaluation (Lord and Corsello 2005). Later, in recognition of the phenotypic variability in autism, the Childhood Autism Rating Scale (CARS; Schopler et al. 1980, 2010), a direct observation standardized measure, was developed to allow clinicians to incorporate ratings of frequency, intensity, duration, and atypicality of a child’s behavior into their assessments. While the instruments described report adequate interrater reliability, their discriminative validity in differentiating diagnoses largely varies depending on study participants and comparison group selected (Cohen et al. 1978; Lord et al. 1989; Parks 1983; Volkmar et al. 1988; Wenar et al. 1986).

Currently, the Autism Diagnostic Observational Schedule, Second Edition (ADOS-2; Lord et al. 2012b) and the Social Responsiveness Scale, Second Edition (SRS-2; Constantino and Gruber 2012) are among the most widely used dimensional measures to quantify autism severity with relative independence from participant characteristics such as IQ (Gotham et al. 2009). The ADOS-2’s calibrated severity scores (CSS) and the SRS-2’s T-scores have been useful tools for measuring the degree of social communication impairments and repetitive behavior patterns (Kim et al. 2018; Wiggins et al. 2019). Dimensional measures such as these can provide information about the phenotypic profiles in autism, both related to autism symptoms as well as non-autism related symptoms, that can contribute to improved treatment planning and better symptom tracking over time (Gotham et al. 2009; Kim et al. 2018).

### Dimensional Approach to Core ASD Symptoms and DSM-5

The dimensional approach to DSM-5 captures the homogeneity of core ASD symptoms with the aim of relatively high specificity, while allowing for heterogeneity in the quantity and quality of these symptoms. For example, while individuals must meet two of the four broad principles within the RRBs domain to receive an ASD diagnosis, individuals can vary on the number of principles met (2–4) and the quality or severity of these impairments. This flexibility was improved through DSM-5’s addition of unusual sensory responses/interests as a principle within the RRBs domain, supported by research about its prevalence in ASD (Billstedt et al. 2007) and its usefulness in differentiating ASD from other disorders (Wiggins et al. 2009). This new principle provides an additional symptom description through which individuals can meet diagnostic criteria.

The quantification of various types of symptoms through using standardized instruments such as the ADOS and SRS has also allowed researchers to identify developmental patterns and predict outcomes. Longitudinal analyses have identified various patterns, including for example an inverse relationship between quantity of RRBs at age 2 and language abilities at age 9 (Anderson et al. 2007). Furthermore, qualitative information about symptoms, facilitated through the inclusion of domain severity levels, can provide additional information about the degree of support needed in identified areas of deficit. A dimensional approach to classifying core symptoms allows for the creation of phenotypic profiles for individuals of various ages and developmental levels, captures the individual variation across the spectrum, and ultimately assists with clinical conceptualizations and treatment planning.

Recognizing the heterogeneous patterns of ASD symptom development, DSM-5 and ICD-11 incorporated a developmental perspective into the age of onset criteria. DSM-5 and ICD-11 replaced DSM-IV’s and ICD-10’s criteria requiring symptom onset prior to 3 years of age with a less restrictive onset during the early developmental period with a caveat that some symptoms may not fully manifest until later in life when social demands exceed capacities. Accordingly, DSM-5 and ICD-11 allow for an individual to meet symptom criteria within each domain currently or by history. These changes reflect the developmental nature of ASD, such that symptoms may not become apparent in some individuals until adolescence or adulthood, in other individuals at around age 2–3, and in other individuals after a period of typical development followed by a regression or a plateau (included in ICD-11 as a diagnostic specifier) in skill development.

The developmental approach to symptom onset also resolves the ambiguous distinction between recognition and onset of symptoms. The age at which caregivers recognize symptoms is often different from the age at which professionals diagnose ASD, which are both often different from the age at which the symptoms may actually emerge (Lord and Jones 2012). Retrospective reports reveal that the length of time between recognition and diagnosis often distorts a caregiver’s recollection of the age of initial symptom onset (Hus et al. 2011), thus obscuring the accuracy of onset reporting in previous editions of DSM and ICD. Taken together, in recognition of the heterogeneity in symptom profile, onset, and expression in ASD, DSM-5 and ICD-11 adopted a developmental perspective to better capture individuals with the disorder while simultaneously acknowledging their variability.

### Inclusion of Non-ASD Symptom Specifiers

DSM-5 and ICD-11 further capture heterogeneity in the phenotypic profiles of individuals with ASD through the recognition of non-ASD symptom specifiers in similar, but different ways. Notably, DSM-5 specifiers are used to qualify ASD diagnoses, while ICD-11 specifiers are used to define the subtypes of ASD and to qualify ASD diagnoses. The inclusion of largely the same specifiers in DSM-5 and ICD-11, including intellectual impairment, language deficits, and psychological and medical co-occurring conditions, improves the diagnostic specificity of ASD, provides more fruitful clinical information to guide treatment planning, and allows for the identification of subgroups within ASD to inform developmental trajectories.

#### Cognitive Functioning

Individuals with ASD have been shown to vary widely in cognitive abilities, from severe intellectual disability to superior intelligence, with individuals in these extremes differing in outcomes (Gillberg 1991; Lord et al. 2006; Sheinkopf and Siegel 1998) and in ASD symptom severity (Lord et al. 2006; Sheinkopf and Siegel 1998). The relationship between IQ and symptom severity across most observational and parent-report measures is generally high (Gotham et al. 2007; Hus et al. 2007). More specifically, individuals with ASD with low nonverbal IQs, compared to those with greater cognitive abilities, show increased repetitive behaviors (Gabriels et al. 2005) and greater social–communication difficulties (Lord and Jones 2012). While the relationship between IQ and symptom severity is unsurprising given individuals with lower cognitive abilities likely possess fewer strategies to compensate for ASD-specific deficits (Lord and Jones 2012), it is important to consider these associations when disentangling the effects of ASD and IQ on phenotypic profiles. Thus, in addition to assessing co-occurring intellectual disability, the degree and quality of intellectual impairment (for example, verbal vs. nonverbal discrepancies, differences between IQs of 20 and 50 or between 80 and 110) must be considered when characterizing the presentation of ASD.

#### Language Abilities

Language impairment is neither specific nor universal to ASD (Baird et al. 2008; Grzadzinski et al. 2013; Hartley and Sikora 2010), though many children with ASD do show delays and/or deficits in this area (Boucher 2012; Matson and Neal 2010; Solomon et al. 2011). Patterns of language development in ASD are also variable, such that many children with language delays during very early childhood become fluent speakers during school years (Smith et al. 2007), while other children never acquire expressive language (Boucher 2012). Language ability has also been linked to outcomes, such that individuals with minimal verbal abilities often show more severe ASD symptoms (Lord and Jones 2012) and greater intellectual impairment (Luyster et al. 2008). Neurobiological findings provide further support for the importance of language profiling in ASD. Structural brain analyses show similar abnormalities in core language regions of the brain between individuals with ASD and co-occurring language impairment and individuals with specific language impairment without ASD, while individuals with ASD without language deficits do not exhibit this pattern (De Fossé et al. 2004; Grzadzinski et al. 2013). One of the intentions of removing severe language delay as one example of a communication deficit in autism was to highlight that language delay and autism are not the same and to encourage clinicians to recognize when a child or an adult has both conditions. Thus, while language impairment is not included in DSM-5 autism diagnostic criteria, it is retained as a specifier, as well as an entirely separate diagnosis, and should be assessed given its influence on ASD phenotypic profiles.

#### Psychological and Medical Co-occurring Conditions

Co-occurring psychiatric and medical disorders are common among individuals with ASD, with estimates suggesting that 63–78% of individuals with ASD have at least one co-occurring psychiatric condition (Simonoff et al. 2008; Strang et al. 2012), and approximately 10–77% have at least one co-occurring medical condition (Muskens et al. 2017; Betancur 2011). The most common co-occurring psychiatric conditions are anxiety disorders, attention-deficit/hyperactivity disorder (ADHD), depressive disorders, and oppositional defiant and conduct disorders (Simonoff et al. 2008), while the most common co-occurring medical conditions include gastro-intestinal problems, sleep difficulties, and seizures (Muskens et al. 2017). DSM-5 and ICD-11 criteria support the inclusion of specifiers to denote the presence of co-occurring psychiatric and medical diagnoses because the interplay of ASD with co-occurring conditions influences clinical presentations, developmental trajectories, treatment planning, and outcomes.

For example, individuals with ASD and co-occurring ADHD, compared to those with ASD alone, typically display a greater severity of autism symptoms, especially within the social domain, increased internalizing and externalizing behaviors (Sprenger et al. 2013), and more repetitive behaviors (Gabriels et al. 2005). Compared to individuals with ASD alone, those with ASD and co-occurring anxiety also demonstrate more severe autism symptoms, in addition to greater impairments in psychosocial functioning more generally (Bellini 2004; Tantam 2000). Co-occurring depression among individuals with ASD, especially among those with low IQs, has been associated with an increase in RRBs (Ghaziuddin et al. 2002). Issues in executive functioning (Corbett et al. 2009) and emotion regulation (Mazefsky et al. 2013) are also increasingly being addressed, though are not yet included as specifiers in the formal diagnostic systems. Finally, the presence of co-occurring gastro-intestinal disturbances, seizures, and sleep problems among individuals with ASD has been associated with more severe behavioral symptoms (Aldinger et al. 2015). These findings suggest that the presence of co-occurring psychiatric and medical conditions may be linked to increased impairment beyond core ASD deficits among individuals with ASD, and the influence of these conditions must be considered in assessment and treatment.

### Subdimensions Within Core ASD Symptoms

As diagnostic criteria for ASD have expanded to account for the heterogeneity in the quantity and quality of core and related symptoms (APA 2013), researchers have attempted to identify subdimensions within the core symptom domains of social–communication and RRBs to improve phenotyping. Using items from the ADOS-2, Autism Diagnostic Interview-Revised (ADI-R; Rutter et al. 2003), and SRS-2, Zheng et al. (2020) established a four substantive-factor model within the social–communication domain that may capture the individual variability in symptoms. The first factor, “basic social communication skills”, included items measuring nonverbal communication, joint attention, emotional expression, and emotion recognition. Support for the “basic social communication skills” subdimension also comes from Bishop et al. (2007), who identified this factor when comparing children with ASD to children with diagnoses other than ASD. The second factor from the Zheng et al. four-factor model was “interaction quality”, which was comprised of items related to the quality of conversations, initiations, and responses. The third factor, “peer interaction and modification of behavior”, included items measuring the quality of peer interactions and the extent to which individuals modify behaviors to interact appropriately with peers. The final factor, “social initiation and affiliation”, consisted of items about play, affiliation, and initiation of social interaction with peers (Zheng et al. 2020).

Factor analyses from widely-used diagnostic instruments have also yielded subdimensions within the RRBs domain. Multiple studies (Bishop et al. 2006, 2013; Cuccaro et al. 2003) have identified two factors that may represent unique phenotypes of RRBs among individuals with ASD: (1) “repetitive sensory-motor behaviors” and (2) “insistence on sameness”. Factor 1 consists of motor mannerisms, sensory seeking behaviors, repetitive use of objects, and more generally self-stimulatory behaviors (Cuccaro et al. 2003; Bishop et al. 2006). While the number and intensity of these behaviors are diagnostically useful in discriminating ASD from non-ASD (Kim and Lord 2010), the number, severity, and persistence of these behaviors across development may be important in identifying subgroups within ASD (Bishop et al. 2006). Factor 2 encompasses behaviors related to compulsions, rituals, and resistance to change (Cuccaro et al. 2003; Bishop et al. 2006). Factor 2 behaviors tend to develop later than factor 1 behaviors and have been shown to be stable over time among individuals with ASD (Bishop et al. 2006).

Interestingly, the “repetitive sensory-motor behaviors” factor has been found to be negatively associated with IQ and age, while the “insistence on sameness” factor has shown no relationship or a slightly positive relationship with IQ and age (Bishop et al. 2006; Richler et al. 2010). Thus, although these factors are significantly correlated, their different relationships with other characteristics including IQ and age (Bishop et al. 2006; Kim and Lord 2010), as well as their different trajectories across development (Richler et al. 2010), may suggest that they represent separate constructs (Bishop et al. 2013). While a third potential subdimension within RRBs, “circumscribed interests”, has emerged in some studies (Lam et al. 2008) encompassing restricted interests and unusual preoccupations, the items within this factor have more commonly been incorporated into the previously described two factors (Bishop et al. 2006). Additionally, a relatively new area of research proposes that behavior inflexibility (i.e., patterns of rigid behavior that contrast with the need to adapt to changing environments) may encompass and measure several of the RRBs observed in ASD, and thus may represent a subdimension within core ASD symptoms (Boyd et al. 2012; Lecavalier et al. 2020).

## Controversies

### Criticisms of DSM-5

Concerns about DSM-5 and its impact began to appear even before it was published. Some of these concerns were more general in nature and concerned the entire process of drafting DSM-5 (Frances 2013), while others more specifically centered on developments relative to autism (Greenberg 2013). Concerns were raised about the decision to base the entire revision process at APA headquarters rather than academic institutions, the overreliance, according to some, on previously-collected data using structured diagnostic instruments, and what seemed to some an overly secretive process. While celebrating some aspects of the new system, particularly the long-awaited name change to Autism Spectrum Disorder, a growing concern developed that, despite the best intentions, the new criteria resulted in a narrower concept than DSM-III-R autism.

This skepticism was fueled by preliminary studies evaluating early drafts of DSM-5 criteria that were different from the finalized published version. The first study using early draft criteria (Mattila et al. 2011) suggested that the new criteria might be less applicable to more cognitively able cases, including those with Asperger’s disorder. This study was quickly followed by a study from McPartland and colleagues (McPartland et al. 2012) that reported results of a data reanalysis of cases from the DSM-IV field trial and reported dramatically reduced diagnostic rates not only in cases with clinical diagnoses of autism, but particularly in those with previous diagnoses of Asperger’s and PDD-NOS. Nearly 80% of the latter two groups appeared likely to lose their diagnostic label and thus potential eligibility for services (McPartland et al. 2012). While these studies highlight the need to evaluate diagnostic rates in new editions of DSM, it is important to note that because the previously cited data were collected using DSM-IV criteria, the studies did not include the options available in the finalized version of DSM-5 and hence are not truly comparable.

Another apprehension arose as studies of toddlers and young children reported a concern that tightening the concept would potentially restrict service access and change the nature of the diagnostic concept (Matson et al. 2012). Subsequent meta-analytic studies (Kulage et al. 2014; Smith et al. 2015), again based on previously collected datasets based on DSM-IV criteria, have generally confirmed these concerns, though studies that included larger and richer datasets (for example, item data from the SRS, ADOS, or ADI that were not restricted to criteria from old DSM checklists) have not (Foley-Nicpon et al. 2017; Huerta et al. 2012; Kim et al. 2014). Given evidence of a very substantial overlap between DSM-5 and DSM-IV diagnoses, the DSM-5 work group had earlier adopted a provision that allowed cases with “well-established” DSM-IV diagnoses to be ‘grandfathered’ into DSM-5 in order to avoid patients having to immediately seek new assessments if they had had existing Asperger’s or PDD-NOS diagnoses. However, this has raised some concerns (Galligan et al. 2013; Ohan et al. 2015).

### Asperger’s Disorder and the Broader Autism Spectrum

Asperger’s disorder and the broader autism spectrum have their own interesting and complex, and to some extent, interrelated, histories. In some respects, Asperger’s original report (Asperger 1944) stood in contrast to Kanner’s earlier (1943) paper. The cases that Asperger described, all boys with marked social difficulties (hence the same word autism), somewhat presaged the awareness over the past decades of the “broader autism phenotype” (Ingersoll and Wainer 2014). This awareness has also reflected the similarly growing awareness of the complexity of the genetics of autism (Rutter and Thapar 2014; Yuen et al. 2019). Until Wing’s review of Asperger’s original paper (Wing 1981), however, there was relatively little awareness of the condition (fewer than 100 studies were published on the topic after Asperger’s paper and before Wing’s clinical description in 1993). Wing herself saw the condition as clearly being part of the autism spectrum (Wing 1981) and her paper became the inspiration for what can only be described as a plethora of differing diagnostic views on the concept (Volkmar et al. 2014), with no fewer than 5 distinctive approaches to Asperger’s disorder emerging (see Wing 1993 for further discussion).

Furthermore, it became clear that the persisting site-specific differences in the diagnosis of the condition had continued despite DSM-IV’s attempt to provide a coherent and unifying view of the concept (Lord et al. 2012a). Indeed, a major meta-analysis of over 50 studies conducted after the concept was eliminated from DSM-5 revealed a marked difference in IQ profiles for cases with Asperger’s disorder as compared to those with autism (Chiang et al. 2014). However, the issue is not whether one can find differences between people recruited as having Asperger’s and those not, but rather the reliability, meaning, and validity of these differences across sites and systems. There is no doubt that some people with autism are very different from others; the question is whether a particular term, such as Asperger’s disorder or PDD-NOS or social communication disorder, is helpful in reliably describing those differences (Foley-Nicpon et al. 2017). Researchers also commonly use the term “broader autism phenotype” to describe an even greater range of behaviors extending out from autism and more prominent in families of children with autism than comparison groups, but it has also not yet been defined in a way that has reached the diagnostic manuals.

### Gender

Gender differences exist in the diagnostic profiles of ASD, though there is far more overlap than separation. Epidemiological studies indicate that ASD is more common among males than females, with a ratio estimate from the 2010 Global Burden of Disease study revealing a ratio of 4:1 (Brugha et al. 2016; Loomes et al. 2017). This ratio varies across studies from 2:1 to 5:1 largely due to ascertainment differences, with estimates from population-wide community samples being slightly lower than estimates from administrative record reviews (Brugha et al. 2018). Lower sex ratios have also been noted among community-identified individuals with moderate to profound intellectual disability (Brugha et al. 2016). Notwithstanding the biological evidence suggesting a male majority in ASD similar to other developmental conditions such as ADHD (Willcutt 2012), there is still reason to suspect that females are missed or delayed in diagnosis more often than males. A United Kingdom population-based study found that girls presenting with similar symptom profiles as boys were less likely to receive an ASD diagnosis (Russell et al. 2011).

This gender inconsistency may reflect (1) disparate sensitivity of diagnostic measures that were primarily normed using male-dominated samples, particularly with regard to lower degrees of severity in repetitive behaviors (Charman et al. 2017; Frazier and Hardan 2017) and perhaps sensory symptoms (Øien et al. 2017, 2018); (2) sociocultural factors that may differentially influence the application of diagnostic criteria (Goldman 2013; Kreiser and White 2014); (3) subtle qualitative differences in girls’ presentations of core autism symptoms; and/or (4) greater protective factors in girls that may allow them to ‘camouflage’ their autistic difficulties to avoid detection at a particular symptom level (Bargiela et al. 2016; Constantino and Charman 2016; Lai and Szatmari 2020), although the validity (Fombonne 2020) and the gender specificity (Frazier and Hardan 2017) of the ‘camouflage’ construct have been challenged. While we have generally assumed that diagnostic criteria/methods are gender neutral (Volkmar et al. in press), we must be vigilant on this issue. For these reasons, diagnostic measures need to continue to place a strong emphasis on the need to interpret behaviors within a particular context (including cultural expectations for gender and possible biologically-based sex differences) and to gather detailed developmental histories to supplement the standardized observations when giving diagnostic impressions.

### Culture

Cultural context is a crucial consideration in the diagnostic process, both in accurately assessing for ASD and in understanding the implications of a diagnosis (Freeth et al. 2014). While standardized instruments allow for reliable diagnoses of ASD across countries (Marlow et al. 2019) and diverse populations (Harrison et al. 2017), clinicians must conduct assessments and interpret results within the cultural framework of the individuals they assess. Within some Asian cultures, for example, index finger pointing to express interest is not a common overture, and thus an absence of this skill during an autism assessment may not be coded by a clinician as a behavioral symptom common to ASD (Zhang et al. 2006). Additionally, in South Africa, for example, some children are taught to avoid playing with amphibians and reptiles as safety precautions. Thus, when administering the Afrikaans ADOS or other versions of the ADOS to children who are uncomfortable playing with frogs (which happens in many places), clinicians may elect to use a toy car in place of the toy frog during the “functional and symbolic imitation task” as a culturally sensitive adaptation (Smith et al. 2017).

Similarly, as Freeth et al. (2014) note, issues such as regulation of eye contact and language differences across cultures might impact usual Western-oriented assessments. For example, in one study of Spanish-speaking families in the U.S. (Vanegas et al. 2016), potential issues were noted in the sensitivity and specificity of diagnostic instruments when parents and children experienced language discordance resulting in Spanish-speaking parents underreporting communication impairment in their English-speaking children. Issues relative to the use of screening instruments in various cultures and across multiple countries have also been noted (Dai et al. 2020; Havdahl et al. 2017; Khowaja et al. 2015; Kimple et al. 2014; Rea et al. 2019; Surén et al. 2019; Windham et al. 2014), and, together with the considerations in assessment, highlight the importance of the clinician’s interpretation of behaviors in the context of what would be socially appropriate relative to culture.

The cultural context in which an individual receives an ASD diagnosis is also important, as it may foster acceptance and access to services (common in the U.S.), or it may be associated with stigma for the individual and the family as a whole. In some African cultures, for example, individuals with ASD and their families are stigmatized because of the belief that ASD results from witchcraft (Gona et al. 2015). Furthermore, among cultures that stigmatize disabilities more generally, an ASD diagnosis in the family can also negatively affect the marriage prospects of siblings and the future of the family given the genetic liability (Divan et al. 2012). Across East Asia, the Middle East, and Western societies, a recent review suggested a strong negative impact of ASD stigma on some caregivers resulting in attempts to hide their circumstances (sometimes the child with ASD as well) to avoid rejection from the community (Papadopoulos et al. 2019). Taken together, while little variation in ASD prevalence between cultures has been reported (Elsabbagh et al. 2012), the above studies highlight the importance of navigating the diagnostic process through a cultural lens.

### Social Class

Research suggests that the identification of ASD, rather than the true prevalence, differs by social class (Elsabbagh et al. 2012). The U.S. reports a consistent pattern of increased ASD prevalence among higher socioeconomic status (SES) families (Baio et al. 2018; Durkin et al. 2010, 2017; Maenner et al. 2020; Pedersen et al. 2012), while European countries report increased prevalence among lower SES families (Delobel-Ayoub et al. 2015; Emerson 2012; Rai et al. 2012). The socioeconomic advantage in the U.S. is likely attributable to increased access to services and higher parental education, while the European findings likely result from their universal access to health care and their lack of economic barriers (Durkin et al. 2017), as well as possible social class differences in the need for a formal diagnosis in order to obtain extra financial or social support (which is not generally available in the U.S.). In the U.S., regional prevalence estimates of ASD similarly differ by SES, with Utah (16% poverty) showing an ASD prevalence approximately four times as large as the estimate in Alabama (23% poverty; Mahjouri and Lord 2012). While the rise in ASD prevalence rates throughout the last 2 decades is largely similar in absolute terms across social classes, the prevalence differences between classes, likely attributable to identification rather than true prevalence differences, has largely remained unchanged (Durkin et al. 2017).

### Race/Ethnicity

While prevalence of ASD likely does not differ across racial and ethnic groups (Fombonne 2003; Maenner et al. 2020), the average age of diagnosis continues to differentiate these groups. In the U.S., African American, Hispanic, and Asian children are more likely to receive a diagnosis at a later age than Caucasian children (Maenner et al. 2020; Mandell et al. 2009; Palmer et al. 2010). This delay in diagnosis among ethnic minorities is also evident in some European (Begeer et al. 2009) and Asian (Davidovitch et al. 2013) countries, though additional research is needed to fully understand this pattern. Notably, African American children in the U.S. experience delays in diagnosis despite early parental concerns, with studies reporting an average delay of 42.3 months between a parent’s first voiced concerns about a child’s development and the age of diagnosis of the child (Constantino et al. 2020).

Delays in ASD diagnosis among minority children in the U.S. also stem from the large number of minority children, compared to Caucasian children, who receive non-ASD diagnoses (ADHD, conduct disorder, or adjustment disorder) prior to receiving formal ASD diagnoses (Magaña et al. 2013; Mandell et al. 2007, 2009). Furthermore, among children in the U.S. with DSM-IV PDDs, the specific PDD diagnosis assigned differed by racial/ethnic membership, such that children with Asperger’s diagnoses were significantly more likely to be Caucasian, as well as significantly less likely to be Hispanic, than children with other PDD diagnoses (Rosenberg et al. 2009). Lastly, while the percentage of children diagnosed with ASD and intellectual disability is higher among African American and Hispanic children compared to Caucasian children in the U.S., African American and Hispanic children are nevertheless diagnosed at a later age on average than Caucasian children (Maenner et al. 2020). The later diagnoses among minority youth often result in delays in intervention services (Tek and Landa 2012), highlighting the need for providing greater access to early diagnostic services to minority communities.

### Autism in Developing Countries

While a recent global burden study reported that 95% of all young children with developmental disabilities live in low and middle income countries (Olusanya et al. 2018), the majority remain undiagnosed (Sun et al. 2019). Furthermore, relatively little research originates from these countries, which results in their underrepresentation in the broader ASD literature (Franz et al. 2017). The low diagnostic rates in poor countries likely stem from the lack of dedicated infrastructure to assist people with ASD (Minhas et al. 2015; Tekola et al. 2016), difficulty obtaining referrals to meet with the limited number of specialists (de Vries 2016; Elsabbagh et al. 2012), and low levels of parental literacy that limit a parent’s ability to understand the disorder and to locate services (de Vries 2016; Samadi and McConkey 2011). Families are often forced to manage the care of an individual with ASD on their own, which often involves enlisting the help of extended family and community members (Divan et al. 2012). Among the lucky families who find an available and appropriate assessment center, the target children may be brought to the clinic by non-parent adults, which limits the quality and quantity of relevant developmental information that can be shared with the specialist. Thus, given the numerous barriers to assessment, the children who ultimately receive ASD diagnoses are often the children with the most significant impairments and complex phenotypic profiles (Kommu et al. 2017).

## Conclusions

Overall, there are many recurring themes in the various diagnostic approaches and systems that have been used to address autism over roughly the past 80 years since Leo Kanner described the first 11 children. Much remains similar to Kanner’s first astute descriptions, though we now have a better understanding of the importance and the frequency of co-occurring disorders, as well as the breadth and developmental nature of the core features of social communication deficits and repetitive/restrictive/sensory behaviors. Challenges remain, including how to better understand sex and gender differences, how to apply what we know in different countries, cultures, and populations, how to learn from these differences, how to best use what we know about the dimensions that significantly impact lives, and how to adapt what are clearly dimensions to fit into a bureaucratic and sometimes political world that calls for categories. Another factor that will clearly change as new versions of DSM and ICD are eventually created will surely be greater inclusion of “autistic voices” and input from people with autism and their families. We know more now than we did years ago, but we still have much to learn and much to improve.
